# Racial/Ethnic differences in emergency department triage assignment among visits for substance use

**DOI:** 10.1371/journal.pone.0329376

**Published:** 2025-08-08

**Authors:** Samantha Goldfarb, Natalie Dix, Austin Spitz, Katelyn Graves, Megan Deichen Hansen, Shermeeka Hogans-Mathews, Jessica Day, Jeffrey Harman

**Affiliations:** 1 Florida State University College of Medicine, Department of Behavioral Sciences and Social Medicine, Tallahassee, Florida, United States of America; 2 Florida State University College of Medicine, Department of Family Medicine and Rural Health, Tallahassee, Florida, United States of America; West Virginia University, UNITED STATES OF AMERICA

## Abstract

**Background:**

The opioid/substance use disorder (SUD) epidemic in the United States has become a public health crisis. Stigma by health care workers towards patients with SUD has been identified as a barrier to treatment. Additionally, racial inequities in wait times and service provision have been found in Emergency Departments (EDs).

**Objective:**

The purpose of this study was to examine the racial/ethnic differences in severity of ED triage assignment among visits for SUD.

**Methods:**

This retrospective study utilized pooled data from the National Hospital Ambulatory Medical Care Survey (NHAMCS) from 2016−20. The dependent variable was the recorded triage level for patients with SUD. The independent variable was patient race/ethnicity. Analyses controlled for variables such as age, sex, and arrival by ambulance. Differences in triage level by race/ethnicity among visits by patients with SUD was assessed via multivariable logistic regression models.

**Results:**

Of the reported 788 SUD-specific ED visits from patients with SUD, 56.0% were non-Hispanic White, 28.6% were non-Hispanic Black, 12.9% were Hispanic, and 2.5% were of another race. Visits by Black patients with SUD had 53% lower odds of being assigned to an immediate/emergent triage level compared to visits by White patients with SUD (OR=0.47, *p* = .025).

**Conclusion:**

We found that visits by Black patients with SUD were associated with lower odds of receiving an immediate/emergent triage assignment compared to visits by White patients with SUD, after adjusting for confounding variables. Our results suggest potential dual stigma in ED care of being Black and having a substance use disorder.

## Introduction

The opioid and substance use disorder epidemic in the United States (US) is a significant public health crisis. Between 2022–2023, the first annual decrease in drug overdose deaths since 2018 was reported (107,538 deaths); however, overdose deaths among Black non-Hispanics and Native Hawaiian/Other Pacific Islander non-Hispanics increased during the same time period. [1,2] Importantly, drug overdose deaths increased by nearly 30% from 2019 to 2020. [3] Additionally, there was a continued 14% increase in overdoses from 2020 to 2021, leading to 14.3 million emergency department (ED) visits and over 106,000 deaths. [4–6] According to a Substance Abuse and Mental Health Services Administration (SAMHSA) report from 2021, one in six Americans aged 12 and over (46.3 million) met the applicable DSM-V criteria for having a substance use disorder (SUD) in the past year, [7] which contributes to high personal, familial, and societal costs. Of the 162 million Americans with employer-sponsored insurance alone (1.4% of whom had SUD), the direct medical cost of substance use in 2018 totaled $35.3 billion dollars. [8]

Patients with SUD are often in critical need of clinical intervention; however, barriers related to negative attitudes or stigma by health care workers towards patients with SUD are well-documented. [9–13] Stigma is defined as the mischaracterization by someone in a position of power towards an individual and/or group for health (e.g., disease-related) or non-health (e.g., race, sexual orientation) reasons through labeling, stereotyping, and isolation. [14] This stigma has been reported by patients with SUD, in particular, as a barrier to treatment, thus contributing to increased patient substance use, delayed treatment initiation, and unsuccessful completion of treatment. [15–18] Indeed, stigma by health care workers (physicians, nurses, staff) is associated with avoidance of primary care and higher ED utilization rates. [19–21] Further, qualitative data gathered in New York City from 25 people who inject drugs indicated that over 70% experienced at least one instance of clinical mistreatment by a medical provider and/or healthcare staff member due to their substance use histories. [22]

When there is an intersection of SUD stigmatization and stigma associated with additional patient demographics, such as race and ethnicity, patients may experience a greater spectrum of barriers to appropriate care services. Racial inequities, for example, have been documented repeatedly within ED triage settings, including Black patients being assigned less urgent triage scores, experiencing longer wait times, and being less likely to receive analgesics compared to White patients. [23,24] Multiple studies found racial disparities in the triage process even after adjusting for sociodemographic factors such as gender and insurance type. [24–29] These disparities persist among patients with mental health and substance use disorder in that non-Hispanic Black patients continue to experience significantly longer wait times in the ED compared to non-Hispanic White patients. [30,31] Inequities in care are concerning as they have been shown to result in poor health outcomes, spanning from inadequate pain management to higher mortality rates. [24,28,32,33]

Given that the ED is often the primary point of care for patients with SUD, it is critical that historically minoritized patients such as non-Hispanic Black, Asian, Native American/ Pacific Islanders are seen and engaged in equitable treatment in a fair and timely manner. While research has shown that Black patients with SUD have been found to experience longer ED wait times than White patients with SUD, the reasons for this are unknown. It has yet to be determined if the interplay between a patient’s SUD diagnosis and his/her/their race/ethnicity further impacts triage level assignment. The purpose of this study is to examine the racial and ethnic differences in ED triage assignment among visits for SUD. If non-White patients with SUD are receiving less urgent triage scores than White patients with SUD after adjusting for sociodemographic factors, it may explain why these patients are waiting longer to receive care. It also may highlight the intersectional identities or dual stigma and bias in emergency care treatment associated with a patient diagnosis of SUD and patient race. Our previous study found racial disparities in ED wait times among patients with SUD even after controlling for triage assignment. [30] The purpose of this study was to determine if, and to what extent, racial and ethnic differences persist in triage assignment among visits for SUD.

## Methods

### Data Source

This retrospective study utilized pooled data from the National Hospital Ambulatory Medical Care Survey (NHAMCS) from January 2016 to December 2020. NHAMCS is a multistage probabilistic sample of ED visits in the United States, conducted annually by the National Center for Health Statistics. Sampled ED visits include noninstitutional general and short-stay hospitals, and exclude Federal, military, and Veterans Administration hospitals. Information collected from the annual survey of EDs includes patient demographics, ED visit characteristics (provider’s diagnosis of patient, patient’s stated reason for visit, services provided, treatment/medication given), and hospital characteristics. This study was exempt from Institutional Review Board approval as NHAMCS is a de-identified, publicly available dataset.

### Measures

This sample consisted of ED visits by adults for whom the patient’s stated reason for the visit was categorized as substance abuse which included the NHAMCS Reason for Visit Codes of 1150.0 (abnormal drug usage), 1150.1 (substance abuse, no additional details), 2321.0 (drug addiction or drug dependence), and 5910.0 (intoxication with drugs) (N = 788). We hereafter refer to this as substance use disorder in adherence with the Diagnostic and Statistical Manual of Mental Disorders (DSM-5-TR) criteria which uses this preferred term over substance abuse.

*Triage Level:* The dependent variable was the recorded triage level of the patient. In order to assess the prioritization and urgency of a patient’s need for care, most EDs in the U.S. use a triage algorithm called the Emergency Severity Index (ESI). [34] The ESI is a five-level triage system ranging from immediate/resuscitation to non-urgent that uses triage nurse clinical judgment and vital signs to evaluate patient needs. [34] Triage categories are obtained from a five-level Emergency Severity Index (ESI) algorithm: 1 (Immediate or resuscitation), 2 (Emergent), 3 (Urgent), 4 (Semi-urgent) or 5 (Non-urgent). The algorithm requires the triage nurse to first determine whether the patient is stable or not (e.g., requires life-saving intervention, is considered high-risk physically/psychologically). If the patient is considered stable, the triage nurse is then asked to determine the expected number of resources the patient needs, and to consider the patient’s vital signs in assigning a higher triage score. A Level 1 triage score indicates that the patient requires immediate, life-saving intervention(s); Level 2 indicates that the patient is in a high-risk situation; Level 3 indicates that the patient has an urgent/stable need that requires several resources; Level 4 indicates that the patient has a less urgent/stable need that requires only one resource; and Level 5 indicates that the patient has a non-urgent/stable need that does not require any resources. [35]

*Race/Ethnicity:* The independent variable is patient race and ethnicity, recorded as non-Hispanic White, non-Hispanic Black, Hispanic, or non-Hispanic race other than White or Black (labeled non-Hispanic other). The non-Hispanic other race category included patients who were Asian, Native American/Pacific Islander, or mixed race.

*Control Variables:* Analyses controlled for pain scale score, age, sex (male, female), arrived by ambulance (yes, no), seen in the past 72 hours in addition to current visit (yes, no), whether the ED had a self-check-in available (yes, no), and whether the visit occurred during 2020, the first year of the COVID-19 pandemic in the United States.

### Analyses

Differences in triage level by race/ethnicity among patients with SUD were assessed using STATA 18.5 SE software. Data analyses were conducted using survey procedures with the NHAMCS sampling weights that allowed for the results to be nationally representative and the standard errors to correctly account for the complex NHAMCS sampling strategy. First, the unweighted sampling characteristics were assessed (Table 1). Then, we calculated the weighted proportion of ED visits among patients with SUD that fall into each triage level by race/ethnicity, not adjusting for control variables (Table 2). Logistic regression models were conducted, adjusting for control variables. The outcome variable of triage assignment was coded as binary to represent visits scored as immediate/emergent (Levels 1 and 2) verses not (Levels 3–5). Odds ratio estimates with 95% confidence intervals and *p*-values were obtained (Table 3).

**Table 1:** Unweighted Sample Characteristics, 2016-2020.

	Frequency *Mean* (n = 788)	Percent (%) *Standard Deviation*
Age (in years)	*38.2*	*13.7*
Sex		
Female	290	36.8
Male	498	63.2
Race/Ethnicity		
Non-Hispanic White	441	56.0
Non-Hispanic Black	225	28.6
Hispanic	102	12.9
Other	20	2.5
Arrived by Ambulance		
Yes	396	49.8
Seen in 72 Hours		
Yes	56	7.1
Self Check-in Available		
Yes	73	9.3

Note: Pooled data from the 2016–2020 National Hospital Ambulatory Medical Care Surveys. Visits are limited to those by individuals with substance use disorder, ages 18 and over.

**Table 2:** Weighted Percentages (95% Confidence Intervals) of ER Visit Triage Levels for Patients with Substance Use Disorder, 2016-2020.

	Level 1 Immediate	Level 2 Emergent	Level 3 Urgent	Level 4 Semi-urgent	Level 5 Non-urgent
Full Sample					
	4.3	37.2	42.5	13.4	2.5
	(2.5-7.5)	(31.6-43.2)	(37.2-48.1)	(9.9-17.9)	(1.3-4.9)
Race/Ethnicity					
Non-Hispanic White	4.9	41.6	38.7	12.2	2.6
	(2.4-9.6)	(34.6-48.9)	(32.0-45.8)	(8.3-17.6)	(0.1-6.6)
Non-Hispanic Black	4.0	25.7	50.7	16.4	3.2
	(1.8-8.6)	(19.2-33.6)	(42.0-59.5)	(9.5-26.8)	(1.1-8.8)
Hispanic	3.7	33.9	44.1	16.5	1.9
	(1.1-11.8)	(22.7-47.2)	(30.7-58.3)	(6.7-35.3)	(0.7-5.0)
Other	–	52.1	47.3	0.7	–
	No obs.	(25.0-78.0)	(21.6-74.5)	(0.1-5.0)	No obs.

Note: Pooled data from the 2016–2020 National Hospital Ambulatory Medical Care Surveys. Visits are limited to those by individuals with substance use disorder, ages 18 and over.

**Table 3:** Adjusted Odds Ratios (95% CI).

	Odds Ratio (95% CI)	p-value
**Race/Ethnicity**		
Non-Hispanic White	Ref	
Non-Hispanic Black	0.47 (0.24-0.91)	0.025
Hispanic	0.89 (0.47-1.67)	0.713
Other	1.46 (0.33-6.46)	0.616
**Pain Scale**	0.80 (0.75-0.87)	0.000
**Age**	1.00 (0.99-1.02)	0.503
**Sex**		
Female	Ref	
Male	0.62 (0.37-1.02)	0.061
**Arrived by Ambulance**	1.54 (0.86-2.75)	0.142
**Seen in 72 Hours**	1.18 (0.32-4.41)	0.806
**Self Check-in Available**	0.44 (0.15-1.23)	0.126
**Visit in 2020**	1.33 (0.61-2.90)	0.466

Note: Pooled data from the 2016–2020 National Hospital Ambulatory Medical Care Surveys. Visits are limited to those by individuals with substance use disorder, ages 18 and over.

## Results

Of the 788 reported ED visits from patients with a SUD diagnosis, 56.0% were non-Hispanic White, 28.6% were non-Hispanic Black, 12.9% were Hispanic, and 2.5% were of non-Hispanic other race. Additionally, about 63.2% were male and 49.8% arrived by ambulance. Table 1 presents the unweighted sample characteristics from the NHAMCS data.

Among the full sample, 4.3% were assigned to Level 1 (immediate), 37.2% to Level 2 (emergent), 42.5% to Level 3 (urgent), 13.4% to Level 4 (semi-urgent), and 2.5% to Level 5 (non-urgent). Distributions of ER visit triage assignment varied by race and ethnicity. Assignment to Level 1 represented 4.9% of visits by non-Hispanic White patients with SUD and 4.0% of visits by non-Hispanic Black patients with SUD. Assignment to Level 2 represented 41.6% of visits among non-Hispanic White patients with SUD and 25.7% of visits among non-Hispanic Black patients with SUD. Assignment to Level 3 represented 38.7% of visits among non-Hispanic White patients with SUD and 50.7% of visits among non-Hispanic Black patients. Assignment to Level 4 represented 12.2% of visits among non-Hispanic White patients with SUD and 16.4% of visits among non-Hispanic Black patients with SUD. Assignment to Level 5 represented 2.6% of visits among non-Hispanic White patients with SUD and 3.2% of visits among non-Hispanic Black patients with SUD. Table 2 presents the weighted percentages with 95% confidence intervals of ER visit triage levels for patients with SUD.

The results show that visits by non-Hispanic Black patients with SUD had 53% lower odds of being assigned to immediate or emergent (Level 1 or 2) triage score compared to visits by non-Hispanic White patients with the same condition, and that it is statistically significant (OR=0.47, *p* = .025). There were no significant differences in immediate/emergent triage assignment between visits by Hispanic patients or non-Hispanic patients of other race and visits by non-Hispanic White patients. Table 3 presents the logistic regression models, which represent the odds of an ED visit being assigned to an immediate/emergent triage score versus not, adjusting for potential confounding variables.

## Discussion

The purpose of this study was to determine if racial and ethnic disparities in ED triage assignment exist among ED visits for SUD. We found that ED visits by non-Hispanic Black patients with SUD reported significantly lower odds of receiving an immediate/emergent triage assignment than visits by non-Hispanic White patients with SUD, after adjusting for potential confounding variables. This is concerning as it points to a potential dual stigma in ED care of being Black and having a substance use disorder. Our prior study found that non-Hispanic Black patients with SUD were more likely to wait longer in the ED than non-Hispanic White patients with SUD after adjusting for potential confounding variables including triage level (though this study used high, medium, low, missing, none versus ESI levels). [30] Previous literature also supports the current study’s findings that non-Hispanic White patients with other medical conditions are receiving more urgent triage assignments than non-White patients, despite non-White patients receiving more involved physician workups, high-acuity resource needs, and/or having a behavioral health flag in their electronic health record. [36–39]

This study found that the biggest differences in assigned ESI levels between non-Hispanic White and non-Hispanic Black patients with SUD occur between Levels 2, 3, and 4, which represents the biggest area for potential bias in the triage system. That is, Level 1 is often an objective, immediate assignment given the need for immediate, life-saving care (e.g., resuscitation). However, the results show that race then becomes a significant predictor of being less likely to be assigned to a lower (more urgent) triage level among patients with SUD. The implications of being assigned a higher (less urgent) triage level are profound – both before admission (e.g., longer wait times) and once admitted (e.g., fewer procedures given, services provided, treatment received). The literature documents issues in emergency medicine related to cognitive bias, such as anchoring bias and premature closure. which contribute to under triaging by physicians. Anchoring bias refers to one’s tendency to rely too heavily on a patient’s initial presentation in making diagnostic decisions. Premature closure refers to one’s tendency to accept a patient’s diagnosis (or make a diagnostic decision) without it being fully verified. [40,41] The findings from this study are concerning and add to the growing body of literature that non-Hispanic Black patients with high-risk chronic conditions such as SUD are experiencing inadequate emergency care through long wait times and lower triage assignment scores. One study found that about one-third of ED visits were mistriaged. [42] The study found that, in particular, Black patients had a 4.6% greater relative risk of over-triage and 18.5% greater relative risk of under-triage than White patients in the ED. [42] In addition, high-risk patients (e.g., taking high-risk medications, recent intensive care unit utilization, greater co-morbidity burden) have a higher relative risk of under-triage than patients without these conditions. [42] Though we did not specifically examine the reasons for this in this study, it is important to acknowledge potential cognitive bias and stigma from hospital staff/nurses/physicians, which prior research has shown to be associated with avoidance of primary health care and higher ED utilization rates, and leaving the ED against medical advice among patients with SUD. [19–21] Of further concern, perception of stigma among patients who inject drugs has been associated with worse mental health and lower quality of life scores. [43]

The ED is often the de-facto source of care for psychiatric and substance use treatment due to a lack of inpatient and outpatient behavioral health clinics in the US. [44] Overcrowding of EDs has been a nationwide problem in the last few decades and the recent COVID-19 pandemic exacerbated this issue by prolonging wait times and limiting care. [45] The triage process in the ED is conducted rapidly and relies primarily on subjective information, such as a triage nurse’s clinical judgment. [23] The triage nurses’ role is challenging given that they must quickly assign an ESI level, often in the absence of a full patient medical history or evaluation. These decisions can in turn have life-threatening consequences (e.g., longer patient wait times, mis-assessed patient stability, and/or fewer procedures or treatments received upon admission due to reported resource needs). Though the triage method aims to quickly sort patients given this nationwide increase in ED visits, it often results in under-triage and suboptimal care among non-Hispanic Black and Hispanic patients, even when presenting with severe injury. [23,28,46] This study suggests racial bias in triage assignment may exist among non-Hispanic Black patients with SUD. Future research should examine whether severity of racial bias in triage assignment differs by having a diagnosis of SUD versus not.

### Limitations

There are limitations worth noting in the current study. Due to retrospective chart reviews used in the NHAMCS data, there may be errors in initial reporting or during chart abstraction. Additionally, it is possible the sample size of visits to patients with SUD is understated. The sample used in this study consisted of ED visits for which the patient was recorded as having substance use disorder in the respective Diagnosis category and their stated reason for the visit was substance use. Therefore, it may exclude visits to patients who do have a SUD but did not report it themselves or have it recorded by the provider. Similarly, approximately 35% of individuals with SUD were missing information for the triage level outcome variable and had to be excluded from the sample. Further, some subgroup estimates had small sample sizes, so their results may not be generalizable to the broader population. Of note, we ran additional analyses that included potential confounding variables related to the patient’s number of chronic conditions and vital signs (including temperature, pulse rate, respiration rate, systolic blood pressure, diastolic blood pressure, and pulse oximeter) at visit. None of the above variables were significantly related to triage assignment; thus, we excluded them from the analyses in order to obtain the maximum sample size. Finally, there are no hospital-level socioeconomic indicators to determine potential impact on triage assignment, race, and SUD.

## Conclusions

The current study highlights that patient race is a significant predictor of triage assignment among ED visits for SUD. In particular, ED visits by non-Hispanic Black patients with SUD reported significantly lower odds of receiving an immediate or emergent triage assignment than visits by non-Hispanic White patients with SUD, after adjusting for confounders. Several practice and policy implications are noteworthy based on the study’s findings. First, it is important to understand the impact of unconscious bias among ED physicians, nurses, and staff. Substance use education and training initiatives for health care workers have been shown to improve attitudes towards patients with SUD, and are inversely associated with having negative attitudes towards patients with chronic conditions. [47–49] The findings from this study emphasize the need to adapt these initiatives to additionally address potential racial bias among patients with SUD. Two, the accuracy and precision of the ESI instrument for triage level assignment is questionable given the subjective clinical judgement involved; therefore, an alternate approach to triage level assignment (or modifications to the ESI) may be warranted. Three, there is an immediate need to address bias and stigma in US EDs in actionable ways. A dual-action plan can be implemented to address both structural- and individual-level stigma. [14] This can be accomplished by directly calling out stigma and by developing triage and treatment protocols to assess for and address inequities. Targeted educational interventions that include cultural competency and cultural humility, and motivational interviewing and communication trainings and that address explicit and implicit bias and stigma can help pave the way to equity for non-Hispanic Black patients with SUDs [50].
